# Lab-on-a-micromotor: catalytic Janus particles as mobile microreactors for tailored synthesis of nanoparticles†
†Electronic supplementary information (ESI) available: Supporting videos and figures. See DOI: 10.1039/c8sc03681k


**DOI:** 10.1039/c8sc03681k

**Published:** 2018-10-01

**Authors:** Marta Pacheco, Beatriz Jurado-Sánchez, Alberto Escarpa

**Affiliations:** a Department of Analytical Chemistry, Physical Chemistry and Chemical Engineering , University of Alcala , E-28807 , Madrid , Spain . Email: alberto.escarpa@uah.es ; Email: beatriz.jurado@uah.es; b Chemical Research Institute “Andrés M. Del Rio” , University of Alcala , E-28807 , Madrid , Spain

## Abstract

Catalytic Janus micromotors encapsulating Cd^2+^ or citrate are used here as mobile microreactors for “on the fly” CdS quantum dot and gold nanoparticle synthesis. Micromotor navigation in microliter “reagent solutions” results in the generation of the corresponding nanoparticles inside the micromotor body with high yield and negligible waste generation. Nanoparticle generation can be attributed to convective diffusion of reagents into the moving reactor body. “On-demand” modulation of nanoparticle size and catalytic activities can be achieved by judicious control of the motion behavior of the microreactor. The use of confined reagents in connection with such enhanced movement allows for efficient operation in very low (less than 800 μL) volumes. The new microreactors developed here hold considerable promise for reactions in aqueous environments for novel synthetic schemes in different sites along with multiplexed capabilities for a myriad of catalytic reactions.

## Abstract 2

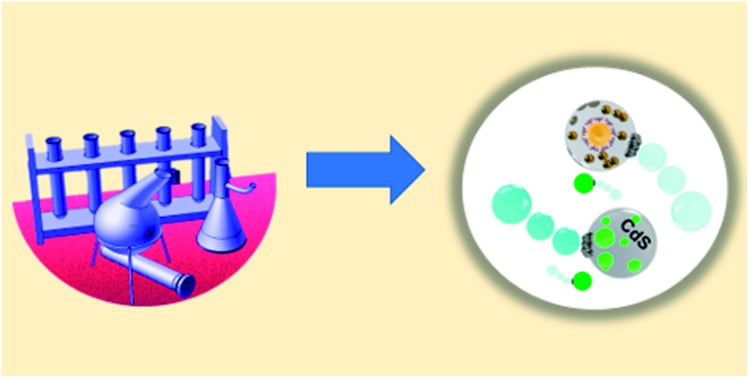
Catalytic Janus micromotors encapsulating Cd^2+^ or citrate are used here as mobile microreactors for “on the fly” CdS quantum dot and gold nanoparticle synthesis.

## Introduction

The ultra-miniaturization of chemical analysis and synthesis processes is a cutting-edge research topic which aims to perform high throughput operations using extremely small amounts of reagents with minimal waste generation. Common synthesis strategies include the use of tiny liquid droplets or microparticles decorated with active nanocatalysts. For example, halloysite,^1^ silica nanotubes,^2^ metal–organic frameworks^3^ or Janus particles^4^ coated with catalytic nanoparticles have been used for *in situ* organic reactions. Silica nanobottles prepared by anisotropic sol–gel growth can be used as vessels for nanoparticle synthesis.^5^ Yet, such configurations are prone to unwanted size reactions and difficult to integrate in ultra-miniaturized environments. As an alternative, the use of microfluidic devices to generate liquid droplets containing adequate additives has been extensively explored to produce noble metal particles, nanocrystals or fluorescent dyes.^6^ Despite its great potential, such technology still suffers from channel-associated cross-contamination and fouling and requires external pumps to drive the fluids and microchannel networks. As an alternative, the so-called liquid “marbles”, or droplets wrapped by a shell composed of hydrophobic magnetic nanoparticles, show considerable potential for small-scale laboratory operations. Indeed, the ability of such tiny droplets to actuate under external forces including magnetic and electric fields has formed the basis for their application in the synthesis of nanocomposites and as “moving” carriers in miniaturized detection systems.^7^

**Fig. 1:**
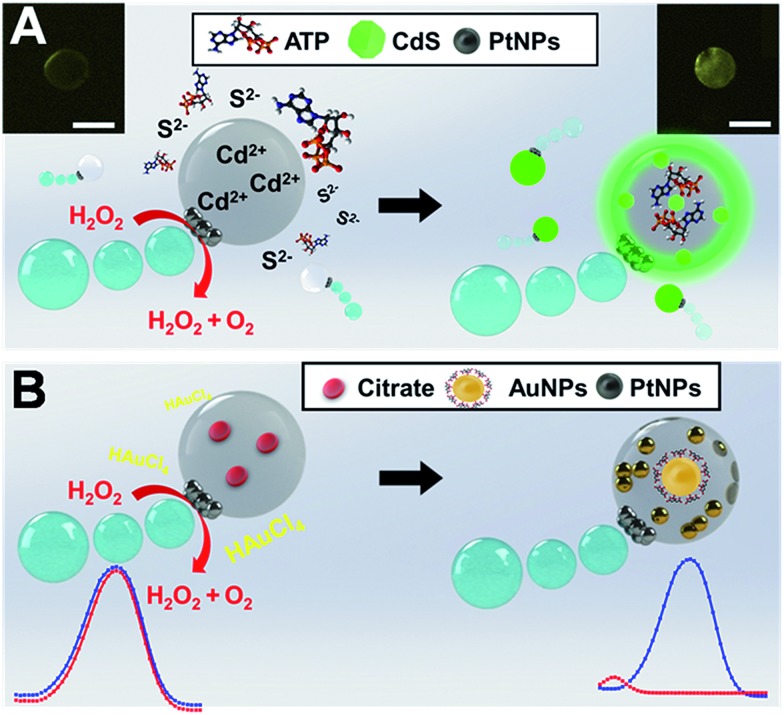
Janus micromotors as mobile microreactors for nanoparticle synthesis. Schematic illustration of the synthesis of CdS quantum dots (top) and AuNPs (bottom) using Cd^2+^ or citrate loaded Janus microreactors navigating in “reagent solutions” containing sulfur ions and ATP or Au^3+^, respectively. The insets at the top (A) show the corresponding fluorescence microscopy images of the micromotors before and after navigation in the reagent solution. The plots at the bottom (B) correspond to the UV spectra of 4-nitrophenol before (blue) and after (red) catalytic reduction by the as-synthesized gold nanoparticles. Scale bars, 20 μm.

Self-propelled micromotors represent another example of small devices capable of performing complex tasks in solution.^8^ Such moving particles can induce efficient fluid mixing and carry active components (“reagents”) in their structure, thus being extremely useful for accelerating chemical operations.^9^ Such capabilities have been exploited in a myriad of environmental,^10^ biomedical,^11^ energy,^12^ and analytical applications.^13^ Value-added product mediated synthesis (vanillyl alcohol) using sodium borohydride propelled graphene based micromotors has also been described.^14^ Wang's group reported the use of micromotors for fabrication of micro- and nano-structures. In a first approach, glucose oxidase modified Au/Ag/Ni flexible nanowires were used for generating 3D Au microstructures with high topological features (*e.g.*, helical shape) on Au patterned substrates.^15^ Later on, metallic nanowires and Janus sphere motors were used as mobile nanomasks and nanolenses to manipulate light beams for generating controlled patterns on a surface in a lithographic like process.^16^ Yet, the application of micromotors as microreactors in ultra-miniaturized synthesis schemes remains unexplored. Herein we report on a new application of Janus micromotors as mobile microreactors for nanoparticle synthesis. The concept, which is illustrated in Fig. 1, relies on cadmium acetate (Cd^2+^) or citrate loaded Janus micromotors for the synthesis of CdS quantum dots and gold nanoparticles (AuNPs), respectively. Micromotor navigation in microliter “reagent solutions” (sulfur ions and adenosine 5′-triphosphate (ATP) or chloroauric acid (HAuCl_4_), respectively) results in the generation of the corresponding nanoparticles inside the micromotor body with high yield and negligible waste generation. Compared with previous studies on micromotors, synthesis can be performed without the need for any enzyme, patterned surfaces or light sources.

The strategy described here is inspired by our recent work on the use of oil-in-water prepared Janus micromotors encapsulating graphene quantum dots for endotoxin detection.^17^ In this work we adopt a similar bottom-up approach to synthesize our moving microreactors, which contain a huge loading of Cd^2+^ or citrate precursors, PtNPs for autonomous propulsion and Fe_3_O_4_ NPs for “on-demand” transport in small settings. Convective diffusion of sulfur ions and ATP or Au^3+^ into the micromotor body triggers the reaction with the precursors facilitating the generation of the nanoparticles (see Fig. 1). To gain further insights into the mechanism behind such nanoparticle generation, we evaluated the effect of different “microreactor” configurations using Cd^2+^ loaded micromotors and S^2–^ solutions and *vice versa*. Similarly, for AuNP synthesis, citrate loaded microreactors in Au^3+^ reagent solutions were used, and *vice versa*. From the results of such experiments, and as will be further described, nanoparticle generation can be attributed to convective reagent diffusion into the moving reactor body. We will also illustrate the “on-demand” modulation of the synthesis conditions in the overall reaction yield and in the catalytic activity of the as-prepared nanoparticles. To the best of our knowledge, this is the first time that self-propelled micromotors have been applied as mobile microreactors in chemistry-based synthesis schemes.

## Results and discussion

**Fig. 2:**
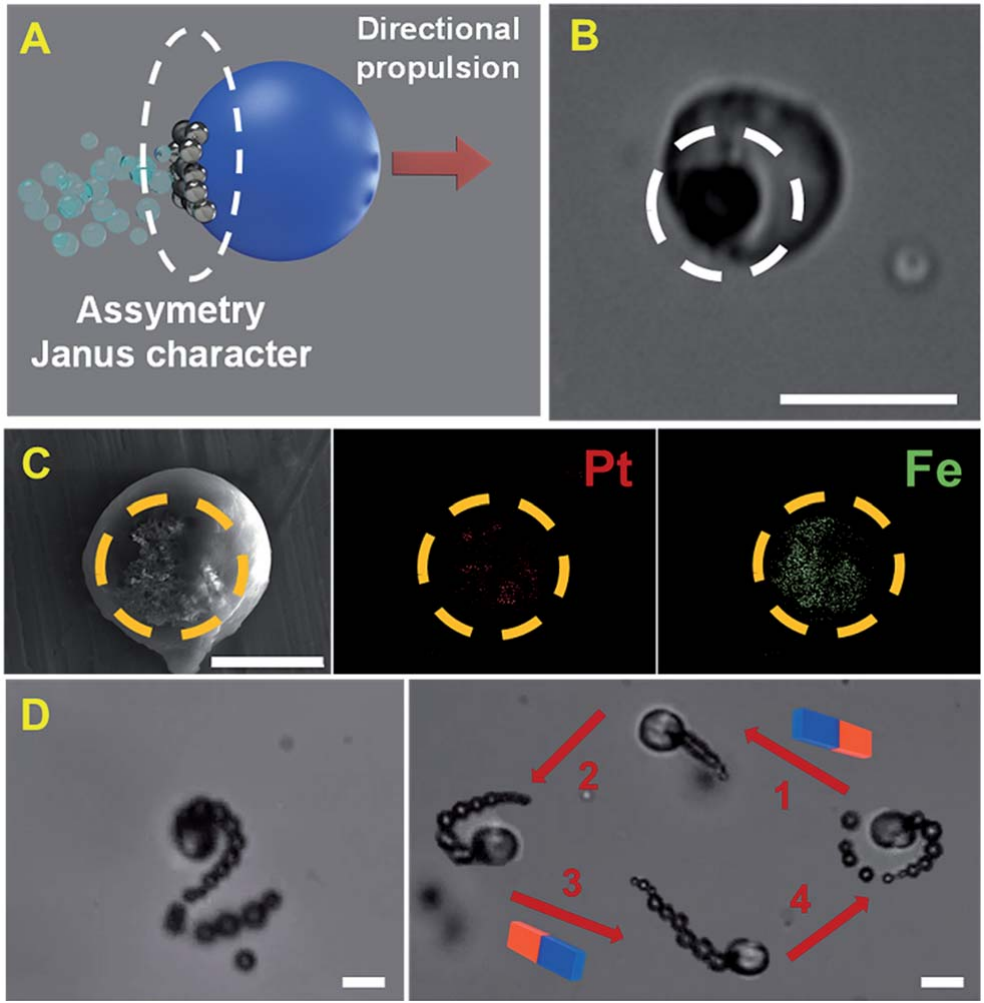
(A) Schematic of the Janus character of the PCL-PtNPs-Fe_3_O_4_ NP microreactor and (B) optical microscopy image of the micromotor. The asymmetric character is marked with a white circle. (C) Scanning electron microscopy (SEM) and energy-dispersive X-ray spectroscopy (EDX) images showing the asymmetric distribution of the catalytic patch in the microreactors. (D) Time-lapse images (taken from ESI Video 1†) showing the propulsion of the microreactor in helical (left) and circular–linear circular (right) trajectories in peroxide solutions and the control of its direction using an external magnetic field. Scale bars, 20 μm.

Fig. 2 shows a schematic and the corresponding characterization of the Janus nature of our microreactor. Following several reports, Janus microparticles can be defined as nano-/micro-objects with asymmetry in their structure. Such asymmetry can thus impart different chemical or physical properties and most importantly directionality within a single particle. Microreactors were prepared using an emulsion polymerization technique. First, an organic solution (chloroform) containing polycaprolactone (PCL), catalytic PtNPs and magnetic Fe_3_O_4_ NPs is mixed with an aqueous 10% sodium dodecyl sulfate solution (please note that nanoparticle precursors are included here). After vigorous stirring, PCL droplets containing Pt and Fe_3_O_4_ nanoparticles inside are generated. Such nanoparticles are not miscible with PCL; thus, during chloroform evaporation they tend to separate from the organic phase and accumulate on the droplet surface to reduce the interfacial energy at the water/oil interface. This results in the asymmetric accumulation of the nanoparticles in one hemisphere of the particle for efficient propulsion/directionality (see Fig. 2A–C). Thus, following the Janus microparticle definition, we define our microreactor as a “Janus microreactor” since the Janus character is introduced by the incorporation of asymmetry/directionality *via* platinum and iron oxide nanoparticle incorporation on one side of the micromotor. As can also be seen in Fig. 2D, such asymmetry induces efficient microreactor propulsion in peroxide solutions, in circular and spiral like motion. Interestingly, the incorporation of magnetic nanoparticles allows for microreactor motion using an external magnetic field for future “on demand” synthesis in ultra-miniaturized environments and other schemes.

**Fig. 3:**
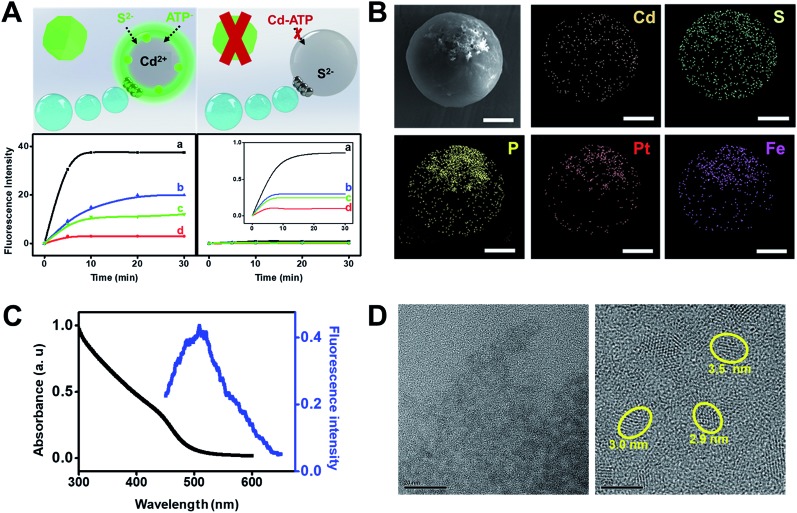
(A) Schematic of the synthesis of CdS quantum dots using Janus microreactors with different configurations. The plots in the bottom part show the fluorescence intensity in the microreactor over time in (a) peroxide solution, (b) under magnetic stirring, (c) with microreactors containing Fe_3_O_4_ in peroxide solutions and (d) under static conditions. (B) SEM and EDX images showing the distribution of Cd, S, P, Pt and Fe in the microreactor. (C) Fluorescent emission and UV/VIS spectra of the as-synthesized CdS quantum dots. (D) HRTEM images of the CdS quantum dots. Scale bars, 5 μm (SEM) and 20 or 5 nm (HRTEM).

Fig. 3 illustrates the synthesis of CdS quantum dots using our Janus microreactors. Two different configurations were explored, one using cadmium acetate dihydrate loaded microreactors (3 × 10^3^ micromotors μL^–1^) in hydrogen peroxide, ATP and Na_2_S solutions and the other using the same amount of Na_2_S loaded microreactors in hydrogen peroxide, ATP and Cd^2+^ solutions. After 15 min, Cd^2+^ microreactors display a strong fluorescent emission at 482 nm after excitation at 340 nm, which was attributed to the generation of the CdS quantum dots. In contrast, no apparent fluorescent emission is noted for S^2–^ loaded microreactors, even after 60 min of navigation in the solutions. From these result, we hypothesized that nanoparticle generation can be attributed to a convective diffusion process into the microparticles.^18^ In Cd^2+^ microreactors, the overall positive charge inside the microreactor body (along with the induced movement) induces the diffusion of oppositely charged ions (S^2–^ and ATP) and the corresponding CdS QD generation. ESI Video 2† illustrates the efficient navigation of the micromotor and the strong fluorescent emission attributed to CdS generation. In contrast, microreactors loaded with Cd^2+^ but navigating in solutions in the absence of S^2–^ do not display fluorescent emission (see the video) even after 30 min of navigation. The synthesis mechanism relies on the use of ATP as a capping ligand, which represents a powerful mean for rationally controlling CdS QD properties. This capping ligand prevents irreversible aggregation by stabilizing the colloid through entropic repulsion, increasing the water solubility.^19^ The low total solution volume (500 μL), which is 4 to 240 times lower than that used in traditional formats,19a,c,^20^ meets the demands of green chemistry principles and holds considerable promise for its use in small settings were mixing constraints can hamper the overall process. Surprisingly, in S^2–^ microreactors, no apparent quantum dot generation is observed. The reason lies in the strong affinity of Cd^2+^ for ATP molecules. Indeed, strong electrostatic interactions between the positive Cd^2+^ ions and the negatively charged oxygen atoms in the phosphate backbones along with the coordination between Cd^2+^ and NH_2_ groups19*b* in ATP result in the generation of a neutral adduct, preventing its diffusion into the microreactor body.

The crucial role of the enhanced fluid mixing generated by the “moving” microreactor toward accelerating the reaction kinetics is illustrated in the time-dependent fluorescence graphics of Fig. 3A. As can be seen, for moving micromotors (a) fluorescence intensity increases rapidly with time up to 5 min of navigation, then reaching a plateau. Please also note that the slope of the graphic in (a) is almost two times higher than the one in (b), indicating the higher yield of CdS. The fluorescence intensity is two times higher than that obtained under magnetic stirring (b), which also reveals a lower yield of CdS quantum dots, further indicating the superior fluid mixing imparted by the micromotors in reduced volumes. For comparison, Fe microreactors in peroxide solutions (c) and static micromotors (d) did not show any apparent increase in the fluorescence intensity, which reveals that the presence of peroxide does not accelerate the synthesis. These results were obtained after analyzing a total of 100 individual micromotor-based reactors, yielding similar fluorescence intensities in all cases. Under the optimized conditions, the as-synthesized CdS quantum dots were further characterized. As can be seen in Fig. 3B, elemental mapping shows a uniform distribution of Cd, S and P in the microreactor body, corresponding to the CdS quantum dots. Also, it can be seen from the fluorescence spectra of Fig. 3C that the CdS quantum dots display a strong fluorescent emission at 482 nm after excitation at 340 nm, which is similar to that reported in other studies for its synthesis.19*a* UV/VIS spectroscopy was used to determine the direct optical band gap according to the Tauc relation,^21^ with the bandgap calculated to be 3.8 eV, yielding a particle size of over 3.6 nm (see the ESI†). The particle size was further confirmed from the HRTEM images of Fig. 3D. Particle analysis of over 50 CdS QDs recorded by HRTEM reveals a mean particle size of 3.6 ± 0.5 nm. Also, the EDX images of Fig. S1† indicate the presence of Cd^2+^ and S^2–^ in the particles.

**Fig. 4:**
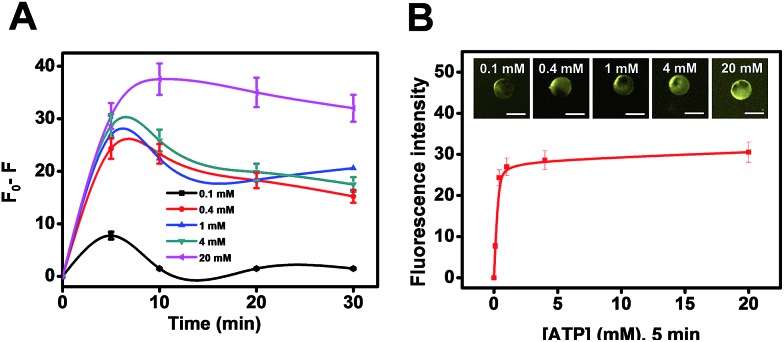
(A) Influence of ATP concentration on the fluorescence intensity of CdS microreactors. (B) ATP detection using CdS microreactors. Scale bars, 20 μm.

Another practical utility of the CdS microreactors is illustrated in Fig. 4. The fluorescence intensity dependence on ATP concentration can be exploited for detection of this important analyte in small settings/ultra-miniaturized environments where common agitation means are not available, revealing its role as a useful microsensor with plenty of capabilities. The maximum signal was obtained after 5 min of microsensor navigation, which was then selected as optimal. Thus, as shown in Fig. 4B, the plot of fluorescence intensity *vs.* ATP concentration displays a linear dependence up to 1 mM concentration and then remains constant. The slight decrease in fluorescence after 15 min was attributed to the aggregation of the CdS quantum dots over time, which can also be partly observed in the HRTEM images of Fig. 3D. Yet, at the optimal ATP concentration, very low aggregation is noted. This strategy offers a convenient way for future sensing profile schemes in different media. Additional efforts in our lab have been aimed at characterizing the CdS quantum dots synthesized under different conditions and extending the concept to synthesize other types of quantum dots, gaining better control of the whole process. On the other hand, the magnetic Fe_3_O_4_ nanoparticles in our microreactor body can be used to selectively transport for controlled “on-demand” release in a pre-determined area, thus opening new and exciting applications in fields such as microfluidics.

**Fig. 5:**
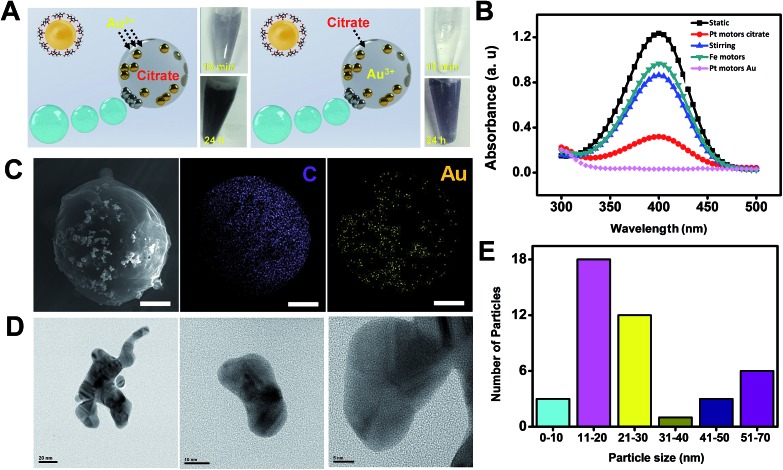
(A) Tailored synthesis of gold nanoparticles using Janus microreactors. The pictures show the resulting colored solution after microreactor navigation for different time periods. (B) UV/VIS spectra of 4-nitrophenol catalytically reduced by AuNPs synthesized under static conditions, with Pt micromotors in bubble solution, under magnetic stirring and with microreactors containing Fe_3_O_4_ in peroxide solutions. (C and D) SEM, EDX and TEM images showing the distribution and morphology of the as-synthesized AuNPs. (E) Plot showing the particle size of the synthesized AuNPs. Scale bars, 5 μm (SEM) and 20, 10 and 5 nm (TEM).

The utility of micromotors acting as highly efficient microreactors was also illustrated for “on-demand” gold nanoparticle synthesis. To this end, 200 μL of a solution containing sodium citrate loaded microreactors (optimal value, 4 × 10^3^ micromotors μL^–1^) was re-dispersed in 200 μL of gold(iii) chloride trihydrate containing 400 μL of H_2_O (for static or magnetic stirring control experiments) or 30% H_2_O_2_ (for bubble propulsion and Fe microreactor control experiments). Similarly, 200 μL of a solution containing gold(iii) chloride trihydrate (optimal value, 4 × 10^3^ micromotors μL^–1^) microreactors was re-dispersed in 200 μL of citrate solution containing 400 μL of the appropriate chemicals. Both microreactors display efficient propulsion in these solutions at a speed of 25 ± 5 μm s^–1^ in the presence and in the absence of a surfactant (see ESI Video 3†). In both configurations, and as can be seen in Fig. 5A, efficient AuNP generation was observed. Yet, for citrate microreactors, fast AuNP generation (which started after 15 min of microreactor navigation) was noted, which can also be visualized by the deep-blue colors of the photograph of the corresponding solutions. This strategy is competitive with conventional approaches. In contrast, 5 h was needed for Au^3+^ microreactors. The slight negative charge of the microreactor surface – due to its preparation in a negative surfactant medium – prevented to some extent the diffusion of negative ions in the microreactor, resulting in slow kinetics.^22^ To demonstrate the quality of the synthesized AuNPs as proof that our approach is viable, we have explored the catalytic reduction of 4-nitrophenol^5^,^23^ using these AuNPs after degrading the micromotor body with lipase (see the Experimental section and Fig. S2 in the ESI† for additional control experiments). The experiments clearly demonstrate that these AuNPs have the expected catalytic properties. From the results plotted in Fig. 5B, nearly 100% degradation of 4-nitrophenol was obtained after the “moving micromotors” experiment (bubble), as compared with magnetically stirred micromotors and static conditions. Such results indicate a higher reaction yield and catalytic activity of the AuNPs associated with the enhanced fluid mixing of the moving microreactors. Additional control experiments using Fe microreactors reveal negligible nanoparticle generation, indicating that peroxide alone is not enough to induce nanoparticle synthesis inside the micromotor.

SEM and HRTEM characterization reveals a uniform distribution of Au in its body, with a variable distribution with most of the particles displaying an average size of 20 nm (Fig. 5E, for more details see also the Experimental section). To further demonstrate the advantages of our micromotor/microreactor, we investigate nanoparticle formation in bulk solution by mixing 5 mM gold(iii) chloride trihydrate, 3% sodium citrate and 15% hydrogen peroxide (total volume, 800 μL) in the absence of microreactors. Gold nanoparticle formation is observed after 5 min of addition; yet, after 30 min particles tend to aggregate and become unstable (see Fig. S3†). In addition, the 4-nitrophenol test revealed the poor catalytic activity of the as-prepared nanoparticles, with no degradation of the compound (not shown). On the other hand, gold nanoparticles remain stable when synthesized using our micromotor approach, which reveals an additional advantage of our procedure over batch synthesis in the presence of high concentrations of H_2_O_2_. Please note here that in experiments conducted with Fe microreactors, no apparent gold nanoparticle formation was observed. This further reveals the microreactor enhanced fluid mixing effect associated with the fast nanoparticle generation.

**Fig. 6:**
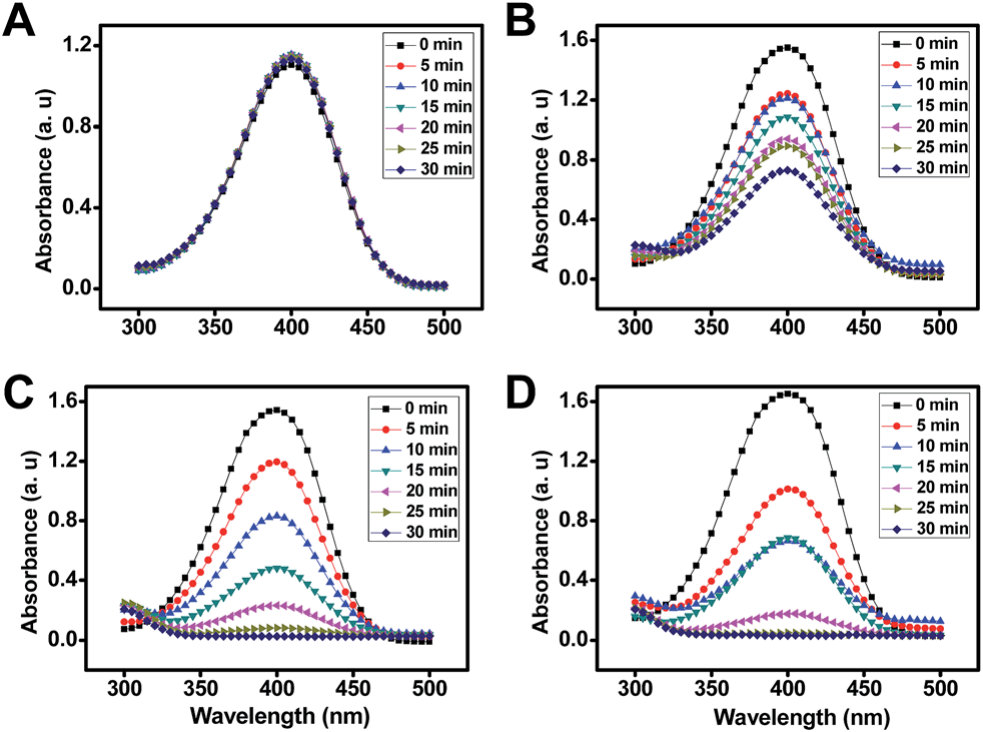
Control of the synthesis rate and catalytic activity of AuNPs using the “moving” microreactors under different experimental conditions: (A) in surfactant-rich media (navigation time, 24 h), (B) Au^3+^ motors at 100 °C (navigation time, 30 min) and (C) Au^3+^ motors (navigation time, 24 h) and (D) citrate motors (navigation time, 30 min) at 25 °C, respectively.

Interestingly, the reaction rate and catalytic activity of the resulting AuNPs can be modulated by the judicious control of the mobile microreactor motion behavior. For example, the addition of a surfactant to the mixture results in the generation of AuNPs with no apparent catalytic activity as illustrated in Fig. 6A. This can be attributed to the passivation of their surface with the surfactant.^24^ Further experiments to evaluate the effect of the surrounding media (propelling environment) reveal that AuNPs synthesized with the mobile microreactors at room temperature (25 °C, Fig. 6C and D) display higher catalytic activity than those synthesized with microreactors navigating in water at 100 °C (Fig. 6B). With this data we evaluate the catalytic performance of the catalytic nanoparticles for the reduction of 4-nitrophenol according to the Langmuir–Hinshelwood model. The apparent rate constant (*K*_app_) was determined from the slope of the linear correlation of ln(*A*_*t*_/*A*_0_) with time, where *A*_*t*_ and *A*_0_ denotes the absorbance at time *t* and at the beginning, respectively (see Fig. S4 in the ESI†).^25^

The reduction of 4-nitrophenol catalyzed by AuNPs synthesized with Au^3+^ motors followed pseudo-first order kinetics, with apparent rate constants of 0.0004 and 0.0035 s^–1^ for the experiments conducted at 100 °C and room temperature, respectively. This corresponds to a particle size of about 100 and 20 nm, respectively. For citrate and Au motors (synthesized at 25 °C) similar apparent rate constants were noted for the synthesized nanoparticles. As can be seen, *K*_app_ decreases as the particle size increases, which provides a convenient means to tailor the morphology and requirements of such nanoparticles for an intended application.

**Fig. 7:**
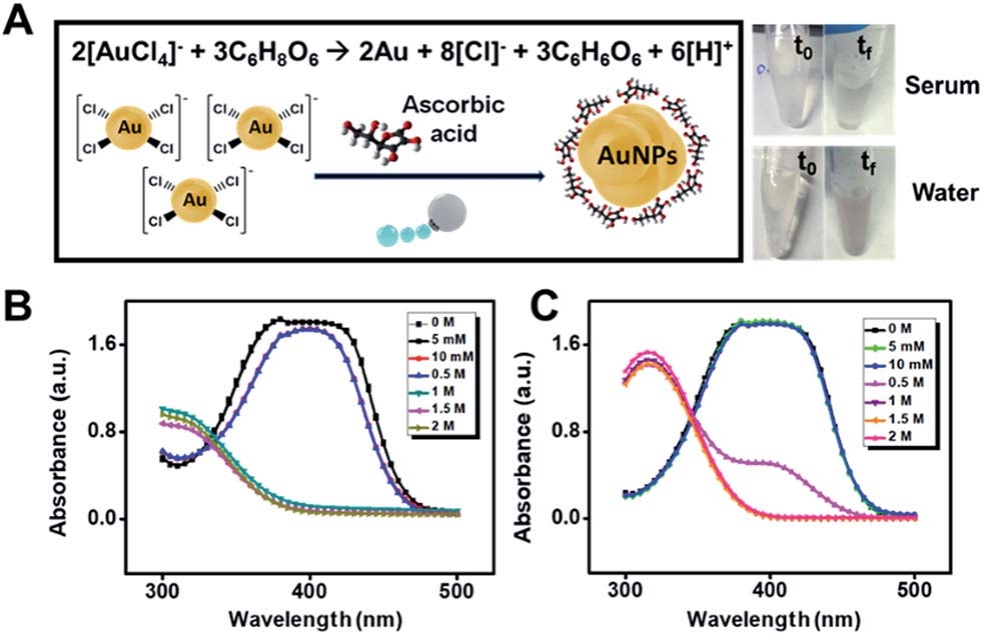
Gold micromotor “reactors” using ascorbic acid as the precursor. (A) Schematic of the ascorbic acid triggered generation of gold nanoparticles. The right part shows the photographs of water and serum solutions containing 2 M ascorbic acid before and after 24 h navigation of the microreactors. (B and C) UV/VIS spectra of 4-nitrophenol catalytically reduced using AuNPs synthesized at different ascorbic acid concentrations in serum and water samples, respectively. *t*_0_ and *t*_f_ correspond to 0 and 24 h, respectively. For details on 4-nitrophenol experiments, see the Experimental section.

Future applications of gold microreactors can be directed to the evaluation of the antioxidant capacity of phenolic compounds and other antioxidants in food^26^ or to surface enhanced Raman scattering (SERS) sensing schemes for the detection of important analytes. In the latter case, the confinement and aggregation of AuNPs inside the microreactors hold considerable promise to create hot spots for enhanced detection.^27^ In this work, to prove the synthesis capabilities of our microreactors, we replace citrate with ascorbic acid, which is an essential antioxidant in food and is also involved in cellular functions. Thus, the assessment of such analytes in intracellular and extracellular environments is relevant for disease diagnosis or to ensure food quality. The concept is shown in Fig. 7. We mix the micromotors (loaded with Au^3+^) with increasing concentrations of ascorbic acid in water and diluted serum samples. As can be seen, after micromotor navigation (24 h) a slight color change is observed in this solution, which is attributed to gold nanoparticle formation following the mechanism depicted in Fig. 7A. To further support such a hypothesis, we treated the motor with lipase and determined the catalytic activity of the as-prepared nanoparticles through the reduction of 4-nitrophenol. As can be seen in Fig. 7B and C, the 4-nitrophenol degradation rate increases along with the concentration of ascorbic acid, indicating a higher yield of nanoparticles at increased concentrations. Such results hold considerable promise for the future application of the micromotor to monitor intracellular events involving ascorbic acid. The concept can also be extended to evaluate the antioxidant capacity of food based on the microreactor triggered gold nanoparticle generation as well as to synthesize tailored AuNPs using natural sources with different properties.

## Conclusions

In conclusion, we have illustrated here, for the first time, the use of catalytic Janus micromotors as “all-in-one” mobile microreactors for nanoparticle synthesis. Cd^2+^ loaded micromotors were used for CdS quantum dot synthesis using ATP as the capping and stabilizing agent. In addition, one practical utility of such an approach is the use of micromotors as microsensors for ATP detection and for controlled transport and release operations. The concept is also applied for AuNP synthesis using citrate or HAuCl_4_ microreactors. The overall nanoparticle size and catalytic activity can be modulated by the judicious control of the motion behavior of the microreactor. The use of confined reagents along with the efficient microreactor navigation allows for a drastic reduction of reagent and waste volumes to less than 800 μL. Also, unlike vessels for gold nanoparticle synthesis that require 24 h for adequate nanoparticle generation,^5^ the moving nature of our microreactors allows for a drastic reduction (to 15 min) of the overall time required (in “citrate” mode). The high catalytic activity of the synthesized AuNPs in the micromotor body can be exploited for future environmental studies on 4-nitrophenol degradation or in precious metal recovery schemes. Additional applications can be directed to the evaluation of the antioxidant capacity in functional foods or in SERS sensing schemes where the confinement and aggregation of AuNPs inside the microreactors hold considerable promise to create hot spots for enhanced detection. Future efforts should be aimed at extending the concept to the synthesis of other nanoparticles and to explore additional polymers as components of the micromotor body for faster degradation and nanoparticle release. To decrease the overall cost, the PtNPs used to propel the micromotor can be replaced by economically viable catalysts such as MnO_2_. In addition, the microreactors can be mass prepared in a cost-effective manner. Overall, the new micromotor based microreactors hold considerable promise to address growing concerns about the impact of chemicals released in the environment and for a myriad of analytical, biomedical and material synthesis applications. Compared with previously developed strategies for miniaturized synthesis,^5^,^6^ the new microreactors developed here represent a “fresh” and fast approach with unprecedented capabilities for their use in microvolume settings where alternative means for agitation are not available. The new microreactors allow for simultaneous mixing, transportation and reaction to accelerate the synthesis of nanoparticles and do not require micropumps or microvalves to drive fluids, avoiding common fouling phenomena. Such a simple synthetic approach allows for controlled synthesis in aqueous environments and in different compartments along with multiplex capabilities for a myriad of catalytic reactions. The moving nature and magnetic properties of the microreactor vessel allow controlled synthesis in ultra-small settings and controlled transport and release in pre-determined settings, with many possibilities that remain to be explored in the near future.

## Experimental

### Chemicals

Polycaprolactone (cat. 440752), iron oxide nanoparticles (20 nm, cat. 700304), chloroplatinic acid hydrate (cat. 398322), hydrazine (30% in H_2_O, cat. 309400), chloroform (cat. 650498), cadmium acetate dihydrate (cat. 289159), sodium sulfide nonahydrate (cat. 208043), Trizma® hydrochloride solution (pH 7.4, 1 M, cat. T2194), adenosine 5′-triphosphate disodium salt hydrate (cat. A26209), lipase from *Pseudomonas cepacia* (cat. 62309), gold(iii) chloride trihydrate (cat. 520918), sodium borohydride (cat. 71320), 4-nitrophenol (cat. 1048), sodium cholate (cat. C9282), hydrogen peroxide (30% solution, cat. 216763) and human serum AB plasma (cat. H4522) were purchased from Sigma-Aldrich (Spain). Sodium dodecyl sulfate (cat. 71727) was supplied by Merck (Germany). Ascorbic acid (cat. 95210) was obtained from Fluka (Spain).

### Instrumentation

Scanning electron microscopy and energy-dispersive X-ray mapping analysis images were obtained using a JEOL JSM 6335F instrument, at an acceleration voltage of 10 kV and 22 kV, respectively. High resolution transmission electron microscopy images were obtained using a JEOL JEM 3000 F microscope at an acceleration voltage of 300 kV. An inverted optical microscope (Nikon Eclipse Instrument Inc. Ti-S/L100), coupled with 20× objective was used to track the speed of the micromotors. Fluorescence images were obtained using an Epi-fluorescence attachment with a UV-2E/C (DAPI) filter cube. UV-VIS experiments were carried out using a Perkin-Elmer Lambda 20 spectrophotometer. Fluorescence spectra were recorded at 25 °C with a PerkinElmer LS-50B luminescence spectrophotometer equipped with a Xe flash lamp. The excitation and emission slit widths were 5 nm and scan speed was 1000 nm min^–1^.

### Microreactor synthesis

Janus microreactors were prepared using an emulsion polymerization technique. Cadmium or S^2–^ microreactors for CdS synthesis were prepared by mixing a 10% sodium dodecyl sulfate solution (8 mL) with 1 mL of cadmium acetate dihydrate (40 mM) or sodium sulfide (0.18 M), respectively. For the gold nanoparticle microreactor, a solution of 5 mM gold(iii) chloride trihydrate (10 mg) or 3% sodium citrate was mixed with the 10% sodium dodecyl sulfate solution. Next, each solution was rapidly mixed with 1 mL chloroform solution containing polycaprolactone (100 mg), platinum nanoparticles (average particle size 100 nm; 5 mg) and iron oxide nanoparticles (20 nm, 200 μL). Fe microreactors were synthesized by mixing the 10% sodium dodecyl sulfate solution containing gold(iii) chloride trihydrate (5 mM) with 1 mL chloroform solution containing polycaprolactone (100 mg) and iron oxide nanoparticles (20 nm, 200 μL). The solutions were vigorously stirred for 10 min and added dropwise onto a Petri dish to improve the evaporation of solvents and polymer solidification. Micromotors were collected in water with a pipette and washed to remove excess reagents at 5000 rpm for 15 min.

### Cadmium sulfide quantum dot and gold nanoparticle synthesis

For CdS synthesis, 100 μL of cadmium or S^2–^-loaded microreactors (3 × 10^3^ micromotors μL^–1^) were dissolved in 100 μL of 0.1 mM Tris–HCl buffer (pH 7.4, for static and magnetic stirring experiments) or hydrogen peroxide solution (final concentration in the mixture, 6%) and mixed with 200 μL of ATP solution. After 5 min, 100 μL of Na_2_S (18 mM) or Cd^2+^ (4 mM) solution was added dropwise into the mixture, and was left to react for 15 min. Next, the fluorescence emission of the microreactors at 482 nm was checked using an optical microscope after excitation at 340 nm.

For AuNP synthesis, 200 μL of a solution containing a known amount of gold(iii) chloride trihydrate loaded microreactors (4 × 10^3^ micromotors μL^–1^) was redispersed in 200 μL of 3% sodium citrate (in H_2_O) or 0–2 M ascorbic acid (in H_2_O or human serum diluted 1 : 2). For reverse micromotor configuration, 200 μL of a solution containing a known amount of citrate loaded microreactors was mixed with 200 μL of Au^3+^ (5 mM). To these solutions were added 400 μL of H_2_O (for static or magnetic stirring control experiments) or H_2_O_2_ (final concentration in the mixture, 15%).

The mixture was retained in the centrifuge tube for 24 h at 25 °C to complete the reaction. This method was compared with the classic synthesis of gold nanoparticles that consists in a reduction chemistry of gold(iii) chloride trihydrate with sodium citrate at 100 °C (reaction time, 30 min). Experiments with solutions containing surfactant (sodium cholate, 5%) were performed in a similar fashion. The generation of gold nanoparticles and their catalytic activity were monitored *via* catalytic reduction of 4-nitrophenol. To this end, 125 μL of aqueous NaBH_4_ solution (100 mM) was added to 1.5 mL of the 4-nitrophenol solution (100 μM) in a quartz cuvette. 1 mL of gold nanoparticles released from the microreactors after their enzymatic degradation was then added to the mixture. To monitor the reaction progress, the mixture was measured at regular intervals of time (5 min) for 30 minutes with UV-VIS spectrophotometry. The average size of gold nanoparticles was determined by analysing over 150 particles from the TEM images using ImageJ software developed by the National Institutes of Health.

Please note that in both cases we carried out experiments to study the influence of peroxide concentration (over the range of 5 to 20%) on CdS and AuNP formation. Similar results were observed along the entire range; thus peroxide does not have any influence on nanoparticle generation. To adjust the optimal concentration of precursors in the final mixture and minimize the use of reagents and subsequent waste, we adjust the amount of peroxide in each set of experiments.

## Conflicts of interest

There are no conflicts to declare.

## Supplementary Material

Supplementary information

Supplementary movie

Supplementary movie

Supplementary movie
