# Supplementary material for: Lab-on-a-micromotor: catalytic Janus particles as mobile microreactors for tailored synthesis of nanoparticles
Source: Chem Sci. 2018 Oct 1;9(42):8056–64. doi: 10.1039/c8sc03681k (PMC6253719; doi:10.1039/c8sc03681k)
Supplement: Supplementary file 1 [file SC-009-C8SC03681K-s001.pdf]

Electronic Supplementary Material (ESI) for Chemical Science.
This journal is © The Royal Society of Chemistry 2018

Electronic Supporting Information (ESI)

Lab-on-a-micromotor: catalytic Janus particles as mobile microreactors for tailored synthesis of nanoparticles

Marta Pacheco[a], Beatriz Jurado-Sánchez[a,b]* and Alberto Escarpa[a,b]*

[a]*Department of Analytical Chemistry, Physical Chemistry and Chemical Engineering, University of Alcala, Alcala de Henares E-28871, Madrid, Spain. E-mail: beatriz.jurado@uah.es; alberto.escarpa@uah.es (Tel.: +34 91 8854995).*

[b]*Chemical Research Institute "Andrés M. del Río", University of Alcala, Alcala de Henares E-28871, Madrid, Spain*

Supporting videos

SI Video 1. Propulsion of PCL-PtNPs-Fe_3O_4 NPs microreactors in peroxide solutions and the control of its direction using an external magnetic field.

SI Video 2. Propulsion and fluorescent emission of the CdS microreactors in peroxide solutions.

SI Video 3. Propulsion of gold nanoparticles microreactors in peroxide solutions in the absence and presence of surfactant.

Supporting figures

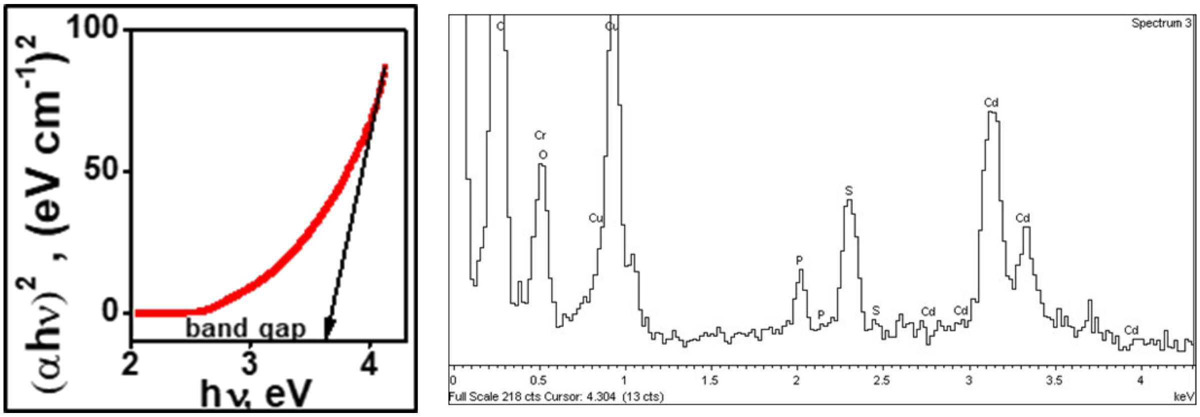

Figure S1. CdS quantum dots characterization: TAUC plot and EDX mapping.

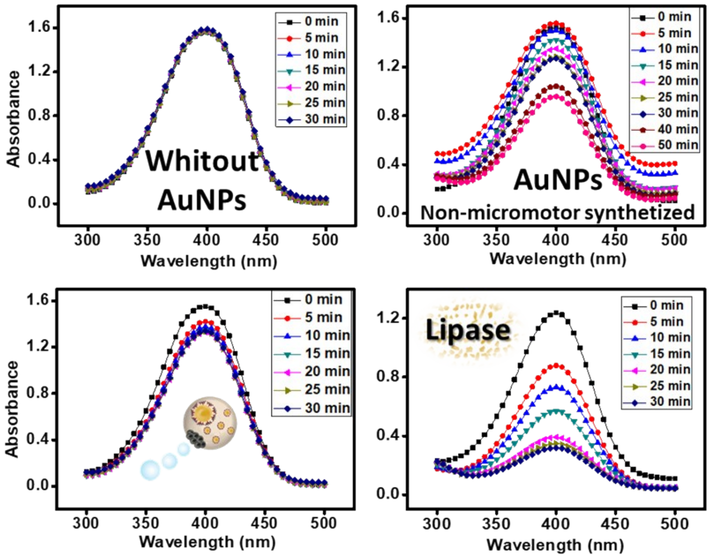

Fig. S2. Tailored synthesis of gold nanoparticles using Janus microreactors: UV/VIS spectra of 4-nitrophenol catalytically reduced by free AuNPs (non-micromotor synthetized), in the absence of gold nanoparticles, using moving micromotors containing the AuNPs and after micromotor degradation with lipase and release of the as prepared gold nanoparticles.

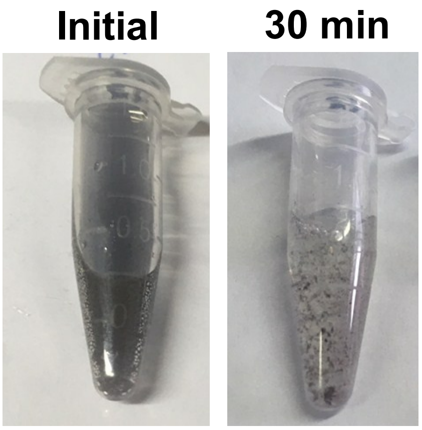

Fig. S3. Photographs of a solutions containing 5 mM gold (III) chloride trihydrate, 3 % sodium citrate and 15 % hydrogen peroxide (total volume, 800 µL) before (left) and after 30 min (right) mixing in the absence of microreactors.

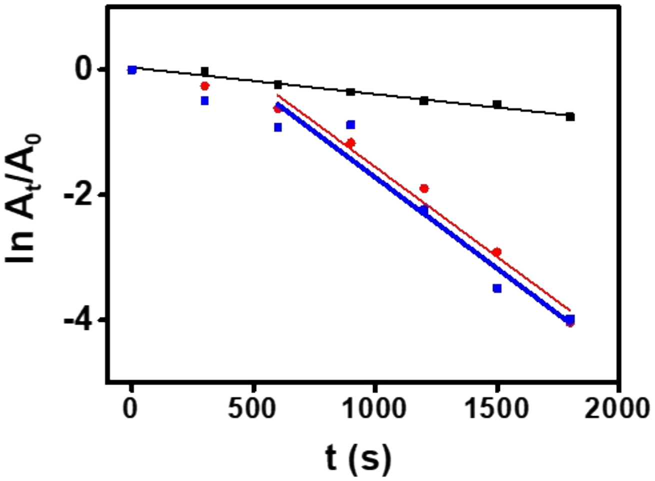

Fig. S4. Kinetic curves of 4-nitrophenol reduction by sodium borohydride catalyzed by AuNPs synthetized with the moving microreactors at 100 ºC (black) and room temperature, 25 ºC (citrate motors, red; Au motors, blue).

Fig. S2 shows several control experiment which reveal the feasibility of the 4-nitrophenol assay to check the catalytic activity and adequate generation of the AuNPs using the moving reactors. Thus, as can be seen in the plot designed as AuNPs, under the presence of such particles, a rapid decrease in the initial absorbance signal of 4-nitrophenol was observed due to its conversion to 4-aminophenol. In contrast, in the absence of AuNPs (whitout AuNPS plot), the absorbance signal remains constant, indication that 4-nitrophenol is not reduced and revealing the crucial role of the AuNPs as catalyst. In addition, plots at the bottom part shows that catalytic activity of the nanoparticles is retained in the microreactor, and that the degradation of the micromotor body with lipase is required to adequately monitor the catalytic activity and charaterize the overall process.
